# Tracheoesophageal fistula after total resection of gastric conduit for gastro-aortic fistula due to gastric ulcer

**DOI:** 10.1186/s40792-017-0371-6

**Published:** 2017-08-23

**Authors:** Yayoi Sakatoku, Masahide Fukaya, Hironori Fujieda, Yuzuru Kamei, Akihiro Hirata, Keita Itatsu, Masato Nagino

**Affiliations:** 1 Division of Surgical Oncology, Department of Surgery, Nagoya University Graduate School of Medicine, 65 Tsurumai-cho, Showa-ku, Nagoya, 466-8550 Japan; 2 Department of Plastic and Reconstructive Surgery, Nagoya University Graduate School of Medicine, 65 Tsurumai-cho, Showa-ku, Nagoya, 466-8550 Japan; 3 Department of Surgery, Shizuoka Kosei Hospital, 23 Kitaban-cho, Aoi-ku, Shizuoka, 420-8623 Japan

**Keywords:** Tracheoesophageal fistula, Gastric conduit ulcer, Pectoralis major muscle flap

## Abstract

**Background:**

Tracheoesophageal fistula (TEF) is a rare but life-threatening complication after esophagectomy. It has a high mortality rate and often leads to severe aspiration pneumonia. Various types of surgical repair procedures have been reported, but the optimal management of TEF is challenging and controversial. Treatment should be individualized to each patient.

**Case presentation:**

A 66-year-old female underwent transthoracic esophagectomy with gastric tube reconstruction and an intrathoracic anastomosis for esophageal cancer. Three years later, she had hematemesis and was diagnosed with a gastro-aortic fistula due to a gastric ulcer. She underwent endovascular aortic repair urgently at another hospital. Two days later, she underwent total resection of the gastric tube, during which time an injury to the trachea occurred; it was repaired by patching the stump of the esophagus to the injury site. Two months later, descending aortic replacement was performed due to infection of the stent graft. Six months after the first operation, a TEF developed. The patient was referred to our hospital for further treatment. The fistula was ligated and divided via a cervical approach, and a pectoralis major muscle flap was used to cover the defect. Esophageal reconstruction with the pedunculated jejunum was performed via a subcutaneous route. The postoperative course was uneventful. The patient was discharged after 6 months of physical and dysphagia rehabilitation.

**Conclusion:**

A TEF located near the cervicothoracic border was successfully treated with a pectoralis major muscle flap through a cervical approach. Total resection of a gastric conduit in the posterior mediastinum carries a risk of tracheobronchial injury; however, if such an injury occurs, surgeons should be able to repair the injury using a suitable flap depending on the injury site.

## Background

Tracheoesophageal fistula (TEF) is a rare but life-threatening complication after esophagectomy. It has a high mortality rate and often leads to severe aspiration pneumonia [1, 2]. Various types of surgical repair procedures have been reported, but the optimal management of TEF is challenging and controversial. Treatment should be individualized to each patient.

Herein, we report a patient with a TEF after total resection of a gastric conduit for gastro-aortic fistula due to a gastric ulcer, successfully repaired with a pectoralis major muscle flap through a cervical approach.

## Case presentation

**Fig. 1:**
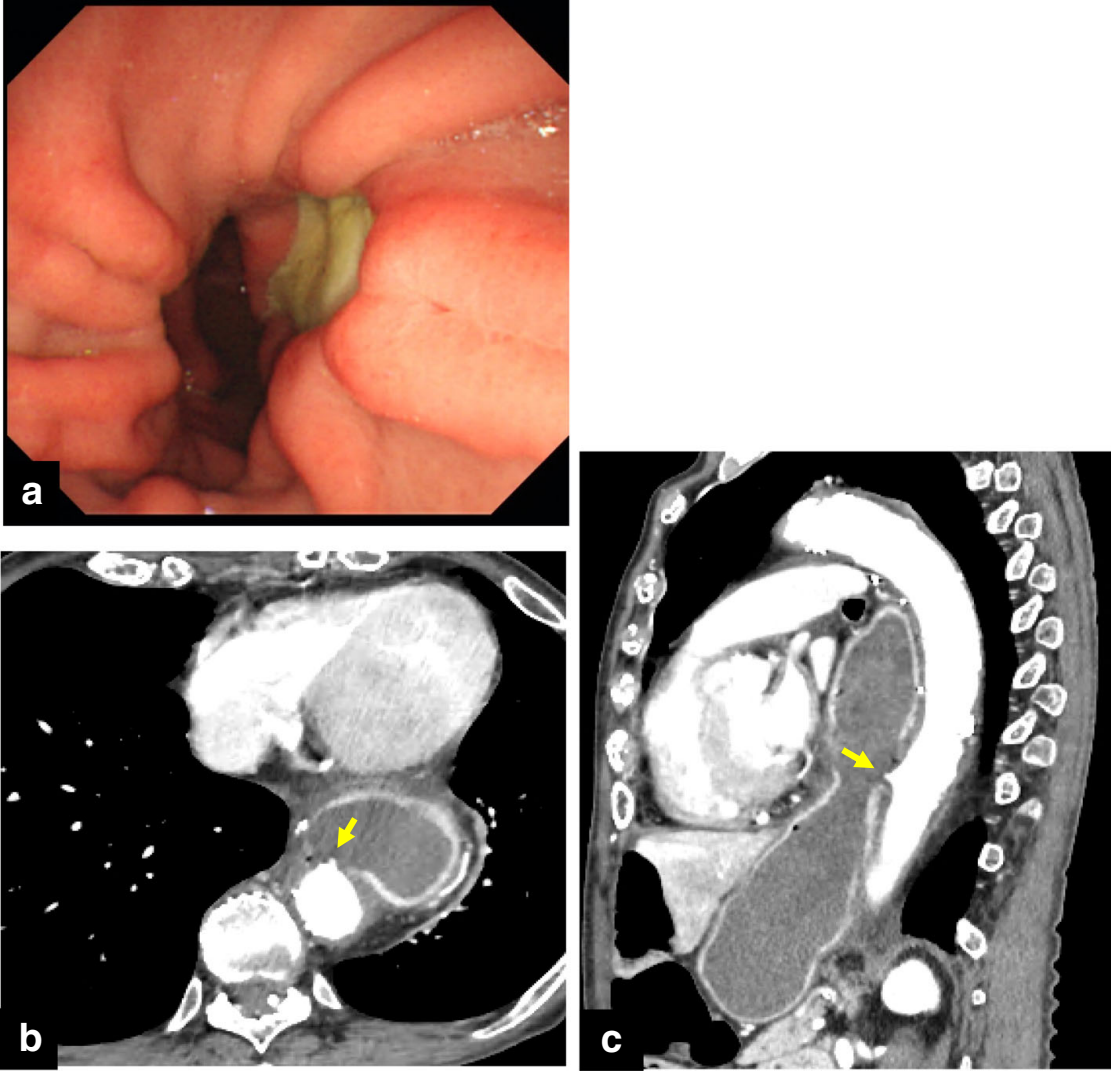
**a** Gastrointestinal endoscopy showed an ulcerated lesion on the right wall of middle of gastric tube. **b** Horizontal and **c** sagittal enhanced computed tomography image showed an irregular ulceration on the anterior wall of the descending aorta, no extravasation, and absence of the descending aortic wall and gastric wall, suggesting sealed rupture of the descending aorta (yellow arrow)

**Fig. 2:**
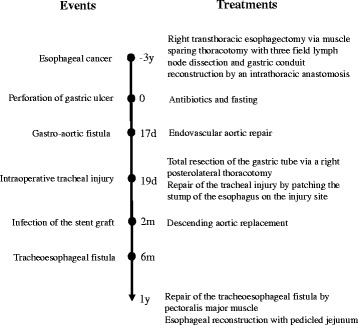
Patient’s clinical course

A 66-year-old woman with esophageal cancer underwent transthoracic esophagectomy with three-field lymph node dissection via a muscle-sparing thoracotomy as previously reported [3], with gastric conduit reconstruction and an intrathoracic anastomosis. A high-dose proton pump inhibitor (PPI) was administered postoperatively due to the patient’s history of gastric ulcers; she discontinued the medication of her own volition. Three years after surgery, she was admitted to a local hospital for mediastinitis due to a perforated gastric ulcer in the conduit (Fig. 1a). She was treated with antibiotics and fasting. Seventeen days later, she had hematemesis and was diagnosed with a gastro-aortic fistula due to a gastric ulcer (Fig. 1b, c). She was transferred to a nearby university hospital with shock status and underwent endovascular aortic repair urgently, using two GORE® TAG® devices (W.L. Gore & Associates, Flagstaff, AZ). Two days later, total resection of the gastric tube was performed via a right posterolateral thoracotomy. There was an abscess cavity between the gastric tube and the descending aorta which consisted of necrotic tissue and old blood. A 2-cm wall defect was found on the right wall of the middle of gastric tube. The clot-filled gastric tube was resected. The perforation of the descending aorta was left. During this operation, a tracheal injury occurred while the remnant esophagus was being separated from the trachea; this injury was subsequently repaired by patching the stump of the esophagus to the injury site. Tracheostomy and a feeding jejunostomy were also performed. The operative time was 8 h and 40 min, and the blood loss was 2380 ml. The remnant esophagus had been decompressed by nasal tube since the total resection of gastric conduit till the following reconstructive surgery. Two months later, a descending aortic replacement was performed due to infection of the stent graft. Stent graft was removed, and the descending aorta between the fifth vertebra and the eleventh vertebra level was replaced with rifampicin-soaked 24-mm J graft (JUNKEN MEDICAL, Tokyo, Japan). Infected vascular intima of the aorta around the gastro-aortic fistula was resected. The operative time was 8 h and 10 min. Purulent matter was found around the stent graft, and *Candida albicans* was recognized by the bacterial culture of the pus. Postoperative severe pneumonia due to methicillin-resistant *Staphylococcus aureus* (MRSA) occurred after aortic replacement and required artificial respirator. Postoperative pneumonia was gradually improved by antibiotics. Three months later, she was transferred to previous local hospital. When a TEF developed 6 months after the first operation, in spite of decompression of the remnant esophagus by nasal tube, she still had required respirator due to prolonged postoperative pneumonia. Her poor general condition could not allow reconstructive surgery. She could withdraw from respirator 5 months after the aortic replacement (7 months after the first operation). Though she suffered from repeated bouts of aspiration pneumonia, she could walk after rehabilitation for 5 months (1 year after the first operation), and transferred to our hospital for reconstructive surgery. This patient’s time course is summarized in Fig. 2.

**Fig. 3:**
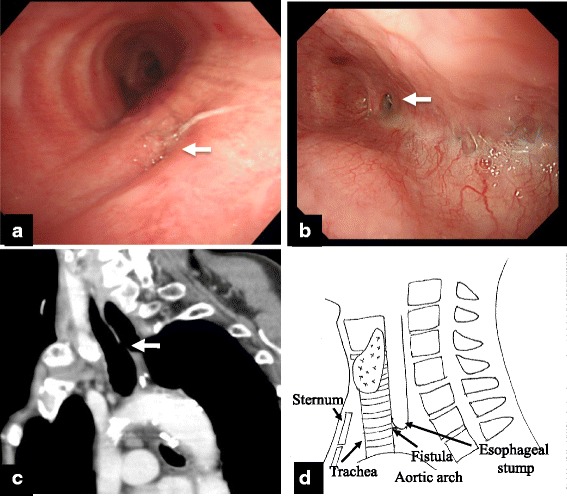
**a** Bronchoscopy showed the fistula on the membranous portion of trachea; a bubble arose from the fistula (white arrow). **b** Gastrointestinal endoscopy revealed a pinhole in the stump of the esophagus and the staple line (white arrow). **c** Sagittal computed tomography image showing the tracheoesophageal fistula on the cervicothoracic border (white arrow). **d** Schema of the tracheoesophageal fistula

**Fig. 4:**
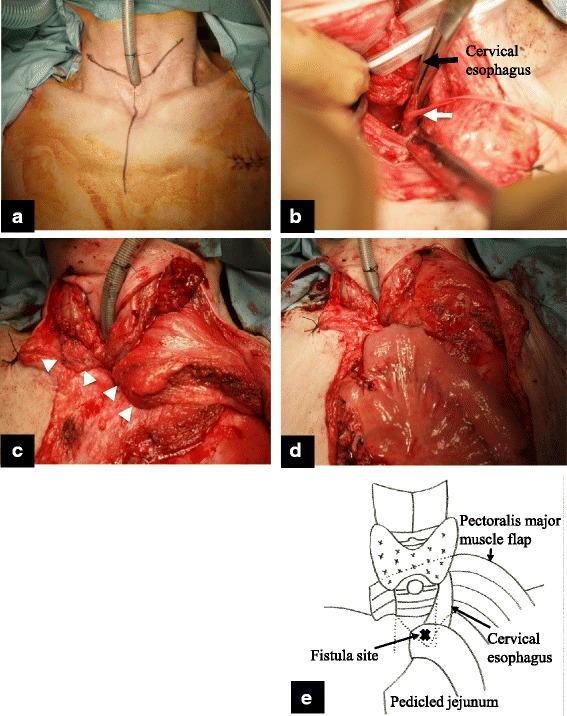
**a** Cervical incision line. **b** The cervical esophagus was accessed through a cervical approach, and the fistula was ligated (white arrow). **c** A pectoralis major muscle flap was fixed to the fistula site, underneath the trachea. Pectoralis major muscle flap under the trachea (white arrow). **d** Esophageal reconstruction using the pedunculated jejunum with a microvascular anastomosis was performed via a subcutaneous route. **e** A schema of the operation

Bronchoscopy showed a fistula on the membranous portion of the trachea (Fig. 3a). Gastrointestinal endoscopy revealed a fistula in the stump of the esophagus (Fig. 3b). Computed tomography showed the TEF to be on the cervicothoracic border (Fig. 3c). The fistula was formed between the trachea and the esophageal stump on the cervicothoracic border (Fig. 3d). The cervical esophagus was accessed through a cervical approach, and the fistula was ligated and divided (Fig. 4a, b). A pectoralis major muscle flap was used to cover the defect and lay under the trachea (Fig. 4c, e). Esophageal reconstruction using the pedunculated jejunum with a microvascular anastomosis was performed via a subcutaneous route (Fig. 4d, e). The total operative time was 8 h and 54 min. Intraoperative blood loss was 453 ml. The postoperative course was uneventful. She was transferred to the previous hospital on postoperative day 14 and was discharged after 6 months of physical and dysphagia rehabilitation.

### Discussion

In patients with TEF after esophagectomy, the latissimus dorsi muscle is the easiest muscle from which to fashion a flap for coverage, but it is often not usable as it is transected in a posterolateral thoracotomy. Though various repair techniques have been reported, the choice of which muscle to use for the repair depends on the location of the fistula. A pedicled posterior pericardial flap or diaphragmatic flap is useful for a fistula located near the carina or near a peripheral bronchus [4, 5]. A pectoralis major muscle flap or sternocleidomastoid muscle flap is useful for a fistula near or in the neck [6, 7]. The intercostal muscle flap can usually reach the whole intrathoracic trachea and bronchus and is a highly versatile option [8].

In this patient, there were several complicating factors including the surgical approach to the TEF given dense adhesions with two prior thoracotomies, flap choice, and conduit choice for esophageal reconstruction. Fortunately, the TEF was located on the cervicothoracic border, and a cervical approach was therefore chosen. A pectoralis major muscle or sternocleidomastoid muscle (SCM) flap can be suitable for repairing this tracheoesophageal fistula. A pectoralis major muscle flap is larger and thicker, and its covering area is wider. A SCM flap can be created easier in the same operative field. The larger pectoralis major muscle flap is considered to be more suitable for this patient to fill the dead space behind the membranous portion of the trachea. There was a problem about blood supply of SCM in this case. The upper third of SCM is supplied by branches of occipital artery. The middle third of SCM is supplied by branches of superior thyroid artery. The lower third of SCM is supplied by branches of the suprascapular artery [9]. There are two types of SCM flap: the superiorly based flap which is the commonly used and supplied from occipital artery and superior thyroid artery and the inferiorly based flap which is suitable for the lesion on the lower neck or upper mediastinum [10]. Though the inferiorly based sternocleidomastoid muscle flap was required according to the fistula position, the necrosis of the flap might occur because of no blood supply from branches of superior thyroid artery and suprascapular artery by previous cervical lymph node dissection. Therefore, the pectoralis major muscle was chosen over the SCM. With respect to esophageal reconstruction, small intestine reconstruction using the pedunculated jejunum with a microvascular anastomosis was chosen over a colonic conduit as there were dense adhesions of the transverse colon to the upper abdominal organs due to a previous total resection of the former gastric conduit.

During gastric conduit resection, dense adhesions around the conduit, particularly around the esophagogastrostomy in the upper mediastinum [11], make dissection difficult and a tracheobronchial injury a possibility. In this case, a tracheal injury occurred while the remnant esophagus was being dissected from the trachea via a thoracotomy. The injury was repaired with the remnant esophagus patch and resulted in a delayed TEF. Because the patient had previously undergone a muscle-sparing thoracotomy for esophageal cancer, thus preserving the latissimus dorsi muscle, a posterolateral thoracotomy with a latissimus dorsi muscle flap or intercostal muscle flap would have been a good option for repair of the initial tracheobronchial injury. When the tracheal injury occurred, the surgeons should have created an intercostal muscle flap to repair the injury and then performed a cervical esophagostomy. A pectoralis major muscle flap or a sternocleidomastoid muscle flap could also have been an option as the injury was near the neck.

The primary cause of this gastro-aortic fistula was discontinuation of PPI medication of her own volition. The frequency of peptic ulcer in the reconstructed gastric tube was reported to be 6.6–19.4% [12–15]. The mortality of patients with perforation of gastric tube ulcer was 56.5% in the review of Japanese literature and 84.6% in the review of the English literature [16]. Once gastric tube ulcer develops to gastro-aortic fistula, it causes a fatal result. We recommend PPI medication for patients with gastric tube reconstruction after esophagectomy.

## Conclusions

 A TEF located near the cervicothoracic border was successfully treated with a pectoralis major muscle flap through a cervical approach. Because total resection of a gastric conduit in the posterior mediastinum carries a risk of tracheobronchial injury, thoracotomy with creation of an intercostal muscle flap should be performed in preparation for a tracheobronchial injury in such situations. If such an injury occurs, surgeons should be able to repair the injury using a suitable flap depending on the injury site.

## Abbreviations

PPI: Proton pump inhibitor
SCM: Sternocleidomastoid muscle
TEF: Tracheoesophageal fistula
